# Hypoglycaemia and treatment patterns among insulin‐treated patients with type 2 diabetes who switched to insulin glargine 300 units/mL versus other basal insulin in a real‐world setting

**DOI:** 10.1002/edm2.73

**Published:** 2019-06-14

**Authors:** Fang L. Zhou, Charlie Nicholls, Lin Xie, Yuexi Wang, Neel Vaidya, Luigi F. Meneghini

**Affiliations:** ^1^ Sanofi Bridgewater New Jersey; ^2^ Sanofi Guildford UK; ^3^ Statinmed Research Ann Arbor Michigan; ^4^ University Of Texas Southwestern Medical Center and Parkland Health & Hospital System Dallas Texas

**Keywords:** Gla‐300, hypoglycaemia, persistence

## Abstract

**Introduction:**

Type 2 diabetes (T2D) is characterized by worsening pancreatic β‐cell function often requiring treatment escalation with oral antidiabetic drugs (OADs), glucagon‐like peptide‐1 and eventually insulin. Although there is much evidence available on the initiation of basal insulins, fewer studies have investigated the effects of switching from one basal insulin to another. This study aims to evaluate treatment persistence and hypoglycaemia in adult patients with T2D on prior basal insulin who were switched to insulin glargine 300 units/mL (Gla‐300) or other basal insulins in a real‐world setting.

**Materials and methods:**

This study is a retrospective cohort analysis of patient‐level data extracted from the Optum^®^ Clinformatics^™^ database between 1 October 2014 and 30 June 2016. Adult patients (≥18 years) with T2D who were being treated with basal insulin during the 6‐month baseline period, who switched to either Gla‐300 or other basal insulins, were followed up for ≥3 months after switching. Outcomes included treatment persistence, and incidence and number of hypoglycaemic events.

**Results:**

Of the included patients, 1204 switched to Gla‐300 and 616 switched to other basal insulins. Adjusting for baseline confounders, patients who switched to Gla‐300 were 34% less likely to discontinue their basal insulin than patients who switched to other basal insulins (hazard ratio [HR] 0.66; 95% confidence interval [CI] 0.54‐0.81; *P* < 0.001). Patients who switched to Gla‐300 were less likely to experience hypoglycaemia at 3‐month follow‐up (odds ratio [OR] 0.56, 95% CI 0.32‐0.97; *P* = 0.039) and at 6‐month follow‐up (OR 0.58, 95% CI 0.38‐0.87; *P* = 0.009) compared with patients who switched to other basal insulins.

**Conclusions:**

Patients with T2D on prior basal insulin in a real‐world setting who switched to Gla‐300 were more persistent with their basal insulin and experienced less hypoglycaemia than patients who switched to other basal insulins.

## 1 INTRODUCTION

Guidelines recommend initiating basal insulin in people with uncontrolled type 2 diabetes (T2D), as reflected by high glycated haemoglobin A1c (A1C) levels, or inability to achieve target A1C levels on oral antidiabetes drugs (OADs) and/or glucagon‐like peptide‐1 receptor agonists (GLP‐1 RAs).^1^, ^2^

New, second‐generation basal insulin analog therapies with improved pharmacokinetic/pharmacodynamic profiles have recently been approved for the treatment of type 1 and type 2 diabetes by the US Food and Drug Administration. Insulin glargine 300 units/mL (Gla‐300) is a formulation of insulin glargine that delivers the same amount of insulin (as Gla‐100) in 1/3 of the volume, which is then gradually released from subcutaneous tissue, resulting in antihyperglycaemic action lasting for 36 hours or more.^3^, ^4^, ^5^ The EDITION series of randomized, controlled trials demonstrated the efficacy and safety of Gla‐300 in a wide range of diabetes patient populations.^6^, ^7^, ^8^, ^9^ In the EDITION 1 and 2 trials, patients with T2D, previously treated with basal insulin, who received Gla‐300, were shown to have similar A1C values but less hypoglycaemia when compared with patients who received insulin glargine 100 U/mL (Gla‐100).^7^, ^9^

There is a growing body of real‐world evidence (RWE) for use of Gla‐300 in patients with T2D.^10^, ^11^ Real‐world data arising from routine clinical care and healthcare service operations, when applied to research questions and analysed using rigorous scientific standards, become RWE and can help in the understanding of how the efficacy and safety data from randomized controlled trials translate in real‐world clinical practice settings and patient populations.

The safety and effectiveness in routine real‐world clinical practice of starting, or switching to Gla‐300, compared with starting or switching to other basal insulins, is becoming increasingly well characterized.^12^, ^13^, ^14^, ^15^, ^16^ Comparisons with first‐generation basal insulins (eg, insulin glargine 100 U/mL, insulin detemir), for example, generally show equivalent improvements in glycaemic control with fewer hypoglycaemic episodes for patients receiving Gla‐300.^12^, ^13^, ^14^, ^15^, ^16^, ^17^ However, despite the increasing amount of real‐world data that are becoming available, there is relatively little real‐world evidence on how well patients persist (remain on insulin during the follow‐up period without discontinuation) on treatment after switching to another insulin.

The focus of this paper is to evaluate treatment persistence and hypoglycaemia in adult patients with T2D on prior basal insulin therapy who were switched to Gla‐300 versus other first‐ or second‐generation basal insulins in a real‐world clinical setting in the USA.

## 2 MATERIALS AND METHODS

### 2.1 Study design

This was a retrospective cohort analysis of patient‐level data extracted from the Optum Clinformatics database between 1 October 2014 and 30 June 2016. The database contains medical claims data, pharmacy claims data and laboratory results, for members of a large US managed care group covering a geographically diverse population across all 50 states of the USA. The database is updated monthly and includes data with service dates from May 2000, comprising approximately 15 million annual covered lives for a total of approximately 47 million unique entries over a 10‐year period.

### 2.2 Patient selection

Eligible patients were adults (≥18 years of age) with T2D who had ≥1 inpatient/emergency room (ER) visit or ≥2 ambulatory medical claims (≥30 days apart) with a primary or secondary diagnosis of T2D (ICD‐9‐CM codes: 250.x0 or 250.x2/ICD‐10‐CM code: E11) during the study period. Patients categorized as long‐acting insulin users were defined with ≥1 basal insulin claim for Gla‐300, Gla‐100, insulin detemir or insulin degludec, during the identification period (between 1 April 2015 and 31 March 2016). The index date was defined as the date of the first Gla‐300 or other index basal insulin claim. Patients were considered eligible, as switching insulin patients, if they had ≥1 claim in the 6‐month baseline period prior to the index date for a different long‐acting insulin, either neutral protamine Hagedorn (NPH), insulin detemir or Gla‐100.

Patients were assigned to cohorts depending on whether they switched to Gla‐300 or another basal insulin (Gla‐100, detemir, degludec) on the index date. Included patients had continuous enrolment in the database with medical and pharmacy coverage for the 6‐month baseline period and ≥3‐month follow‐up period after the index date; they were followed up until disenrolment, death or study end. All patients had ≥1 measurement of A1C level during the baseline period.

### 2.3 Outcome assessments

The study endpoints included treatment persistence, and incidence and number of hypoglycaemic events. Patients were defined as “persistent” if they remained on the index insulin during the follow‐up period without discontinuation after the index date. Patients were considered to have discontinued basal insulin if no claim was made for a refill within the expected time of medication coverage based on the metric quantity of that prescription, defined as the 90th percentile of the time between the first and second fills among patients with at least 1 refill and same metric quantity for the first fill. If no refill was claimed within that period, the date used for discontinuation was the estimated run‐out data of the last claimed refill.

The incidence and number of events (adjusted for baseline confounders) of hypoglycaemia were identified by ICD‐9‐CM/ICD‐10‐CM codes for medical claims in inpatient, outpatient or ER settings during 3‐ and 6‐month follow‐up.

In a subgroup of patients who had A1C measurements at baseline and during 3‐ to 6‐month follow‐up, change in A1C from baseline to follow‐up (latest available value during follow‐up) was also analysed (Tables S1‐S3).

### 2.4 Statistical analyses

Numbers and percentages are reported for categorical variables. Mean values and standard deviations are reported for continuous variables. *t*‐Tests and Pearson chi‐square tests were used to test statistically significant differences between patients who switched to Gla‐300 versus other basal insulins. Cox regression models were used to assess treatment persistence (time to discontinuation), with adjustment for baseline demographic/clinical confounders. Hazard ratios (HRs) are reported for comparisons between treatment groups. Baseline confounders included demographics (age, gender, race, payer type, region) and clinical characteristics (A1C, antidiabetes medication use, comorbidities and diabetes complications including Charlson Comorbidity Index [CCI] score and incidence of outpatient hypoglycaemia). Logistic regression and generalized linear regression models, adjusted for baseline confounders, were used to assess hypoglycaemia incidence and number of events, respectively. Odds ratios ([ORs], incidence) and adjusted means (number of events) are reported. A generalized linear regression model, adjusted for baseline confounders, was used to assess A1C change, and adjusted means are reported. Baseline confounders included demographics (65‐74 and ≥75 years of age, gender, commercial/Medicare payer, race, location), clinical factors (CCI, peripheral vascular disease, liver disease, neuropathy, baseline basal insulin use, baseline basal insulin daily average consumption (DACON; units/d), baseline A1C, ER hypoglycaemia and healthcare utilization, including inpatient stay, ER stay and endocrinologist visit. Baseline confounders were chosen after stepwise selection for multivariable analysis.

## 3 RESULTS

### 3.1 Patient baseline characteristics

**Figure 1:**
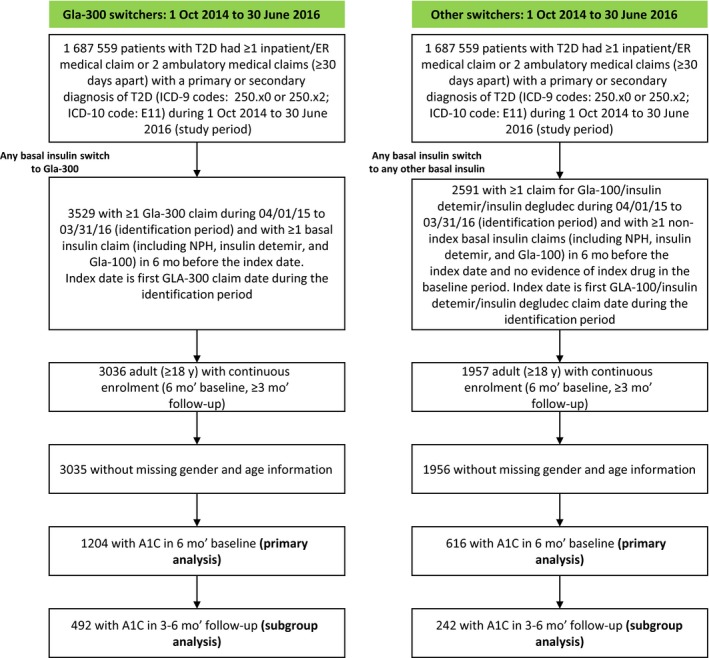
Patient selection

This analysis included 1204 patients who switched to Gla‐300 and 616 patients who switched to other basal insulin analogs (Figure 1).

At baseline (Table 1), patients who switched to Gla‐300 compared with other basal insulins were older (mean 66.7 vs 64.3 years, respectively; *P* < 0.001); were more likely to be on Medicare Advantage plans (88.4% vs 61.9%; *P* < 0.001); had a lower mean CCI score (3.1 vs 4.2; *P* < 0.001); had higher mean basal insulin DACON (41.6 vs 34.1 units/d; *P* = 0.001); were more likely to have used a GLP‐1 RA (13.9% vs 6.3%; *P* < 0.001); had experienced fewer hypoglycaemic events (10.2% vs 13.5%; *P* = 0.038); and were less likely to have had an inpatient stay (11.8% vs 28.7%; *P* < 0.001) or ER visit (25.0% vs 36.9%; *P* < 0.001), but were more likely to have had an endocrinologist visit (29.0% vs 20.8%; *P* < 0.001). Mean baseline A1C levels were comparable between the 2 treatment groups.

**Table 1:** Baseline characteristics

Baseline characteristics	Gla‐300 switchers		Other switchers		Comparing Gla‐300 switcher vs other switchers	
	N = 1204		N = 616			
	N/Mean	%/SD	N/Mean	%/SD	*P* Value	Standardized difference
Age, mean, years	66.65	10.58	64.34	13.24	<0.001	19.28
Gender						
Male	575	47.76%	282	45.78%	0.424	3.96
Female	629	52.24%	334	54.22%	0.424	3.96
Health plan type						
Indemnity	0	0.00%	2	0.32%	0.048	8.06
POS	93	7.72%	165	26.79%	<0.001	52.09
HMO	515	42.77%	259	42.05%	0.766	1.47
PPO	75	6.23%	32	5.19%	0.375	4.46
EPO	22	1.83%	36	5.84%	<0.001	21.02
Others	499	41.45%	122	19.81%	<0.001	48.27
Payer type						
Commercial	140	11.63%	235	38.15%	<0.001	64.40
Medicare	1064	88.37%	381	61.85%	<0.001	64.40
CCI	3.21	2.28	4.22	2.46	<0.001	42.34
OADs						
Any OAD	759	63.04%	355	57.63%	0.025	11.07
No. of OADs	1.03	1.00	0.92	0.97	0.023	11.30
Biguanide	488	40.53%	251	40.75%	0.930	0.44
DPP‐4 inhibitors	143	11.88%	66	10.71%	0.462	3.67
Meglitinide derivatives	16	1.33%	8	1.30%	0.957	0.27
Sulfonylureas	273	22.67%	151	24.51%	0.380	4.33
Thiazolidinediones	51	4.24%	14	2.27%	0.033	11.07
α‐glucosidase inhibitors	5	0.42%	1	0.16%	0.373	4.71
SGLT2 inhibitors	99	8.22%	37	6.01%	0.089	8.62
GLP‐1 RA	167	13.87%	39	6.33%	<0.001	25.20
Baseline basal use (closest to the index date)						
NPH	33	2.74%	318	51.62%	<0.001	131.39
Insulin detemir	308	25.58%	80	12.99%	<0.001	32.32
Gla‐100	863	71.68%	218	35.39%	<0.001	78.06
Baseline A1C values, %	8.94	1.80	8.89	1.92	0.623	2.41
Baseline BI DACON, units/d	41.62	40.53	34.09	46.72	0.001	17.21
Baseline hypoglycaemic events						
Any hypoglycaemia	123	10.22%	83	13.47%	0.038	10.09
Any inpatient/ER hypoglycaemia	28	2.33%	36	5.84%	<0.001	17.83
Any outpatient hypoglycaemia	107	8.89%	69	11.20%	0.114	7.70
Baseline healthcare utilizations						
Any inpatient stay	142	11.79%	177	28.73%	<0.001	43.08
Any ER visit	301	25.00%	227	36.85%	<0.001	25.84
Any endocrinologist visit	349	28.99%	128	20.78%	<0.001	19.06

Abbreviations: CCI, Charlson Comorbidity Index; DACON, daily average consumption; DPP‐4, dipeptidyl peptidase‐4; EPO, exclusive provider organization; GLP‐1, glucagon‐like peptide 1; HMO, health maintenance organization; NPH, Neutral Protamine Hagedorn; OAD, oral antidiabetes drug; POS, noncapitated point of service; PPO, preferred provider organization; SGLT2, sodium glucose co‐transporter 2.

Basal insulin use during baseline differed between patients who switched to Gla‐300 and those who switched to another basal insulin, with Gla‐300 switchers more likely to have been using Gla‐100 (71.7% vs 35.4%; *P* < 0.001) or insulin detemir (25.6% vs 13.0%; *P* < 0.001), but less likely to have been using NPH (2.7% vs 51.6%; *P* < 0.001) in baseline (Table 1). The percentages of other BI users after the switch were 42.1, 57.6 and 0.3 using Gla‐100, detemir and degludec, respectively.

In the subgroup of patients with A1C measurements at baseline and during 3‐ to 6‐month follow‐up (492 switched to Gla‐300; 242 switched to other basal insulins), baseline demographics and clinical characteristics were similar to the overall cohort.

### 3.2 Treatment persistence

**Figure 2:**
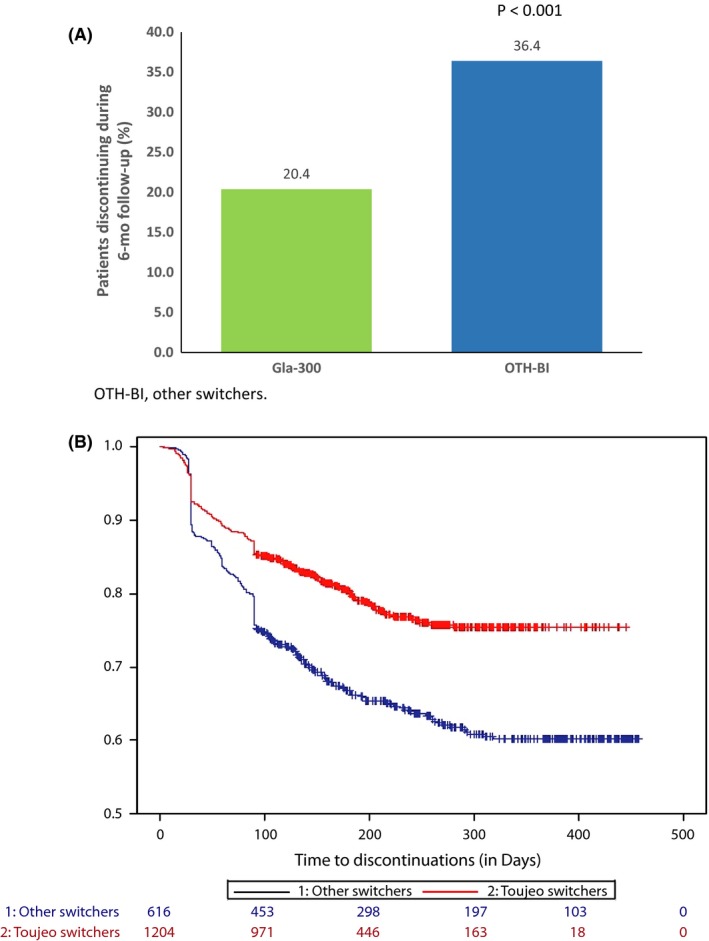
A, Prevelence of treatment discontinuation; B, discontinuation trajectory. OTH‐BI, other switchers

**Figure 3:**
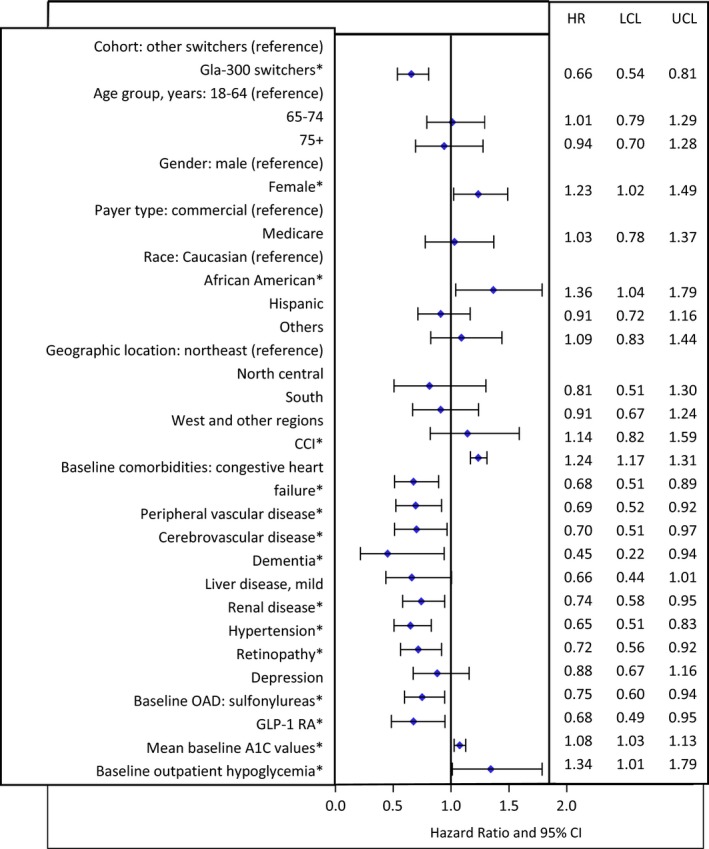
Hazard ratio for risk of treatment discontinuation^a^. ^a^Cox regression analysis for effect of cohort adjusted for baseline confounders. *Baseline confounders with *P* < 0.05. LCL, lower control limit; UCL, upper control limit

Fewer patients who switched to Gla‐300 (20.4%) discontinued their insulin prescription compared with patients who switched to other basal insulins (36.4%) during the 6‐month follow‐up (Figure 2A,B). After adjusting for baseline confounders, Gla‐300 switchers were found to be 34% less likely to discontinue basal insulin therapy during follow‐up compared with other basal insulin switchers (HR 0.66, 95% confidence interval [CI] 0.54‐0.81; *P* < 0.001). The baseline confounders that were associated with persistence or treatment discontinuation included (Figure 3) female gender, African American race, higher CCI, higher baseline A1C levels, and presence of outpatient hypoglycaemia at baseline (associated with increased risk of treatment discontinuation), use of a GLP‐1 RA or sulfonylurea at baseline and baseline comorbidities except for mild liver disease and depression (associated with an increased likelihood of treatment persistence).

### 3.3 Hypoglycaemia

**Figure 4:**
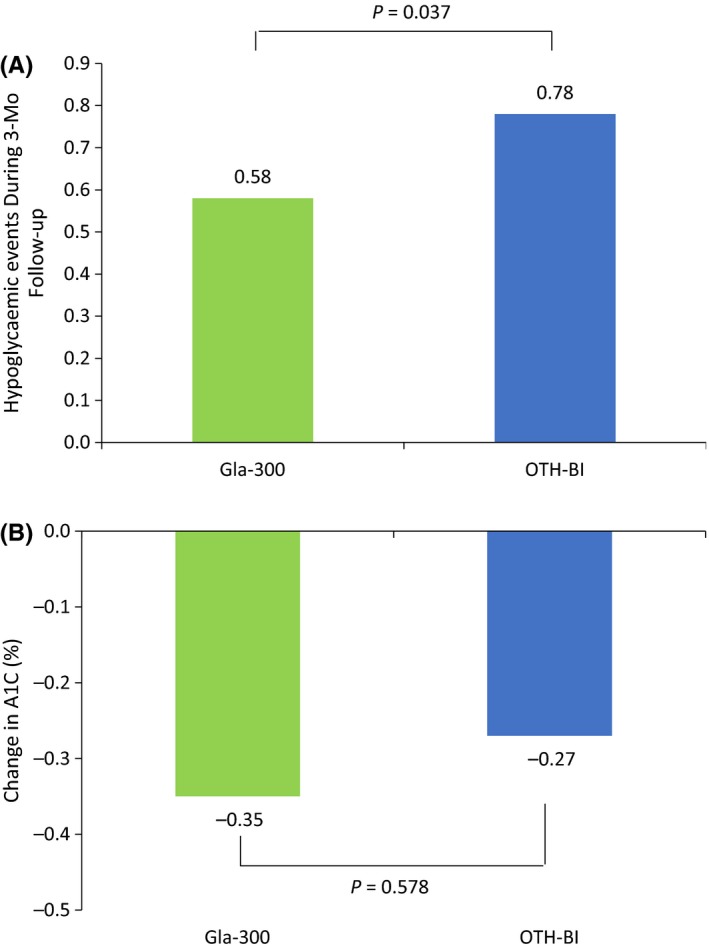
Adjusted hypoglycemic events A, and A1C rate B, during 3‐mo follow‐up. OTH‐BI, other switchers

Fewer Gla‐300 switchers experienced hypoglycaemia at 3‐ and 6‐month follow‐up (Gla‐300 switchers vs other basal insulin switchers: 3 m, 4.4% vs 8.4%, *P* < 0.001; 6 m, 6.0% vs 11.4%, *P* < 0.001). After adjusting for baseline confounders, patients who switched to Gla‐300 were less likely to experience hypoglycaemia at 3‐month follow‐up (OR 0.56, 95% CI 0.32‐0.97; *P* = 0.039) and at 6‐month follow‐up (OR 0.58, 95% CI 0.38‐0.87; *P* = 0.009) compared with patients who switched to other basal insulins. Patients who switched to Gla‐300 had significantly fewer hypoglycaemic events at 3‐month follow‐up compared with patients who switched to other basal insulins (adjusted mean: 0.58 [Gla‐300] vs 0.78 [other basal insulin]; *P* = 0.037) (Figure 4A).

The logistic regression analysis for the 3‐month follow‐up data showed that the presence of nephropathy (OR 1.656 [95% CI 1.036‐2.648]), retinopathy (OR 1.644 [95% CI 1.036‐2.607]), depression (OR 2.481 [95% CI 1.020‐6.031]) and more hypoglycaemic events at baseline (OR 1.091 [95% CI 1.061‐1.122]) were factors associated with increased odds of discontinuation during the follow‐up period, while baseline OAD use was associated with a decreased risk (OR 0.483 [95% CI 0.303‐0.771]).

### 3.4 Change in A1C

In the subgroup of patients who had A1C measurements at baseline and during 3‐ to 6‐month follow‐up, mean reductions in A1C, adjusted for baseline confounders, were modest but comparable between patients who switched to Gla‐300 and other basal insulins (−0.35% vs −0.27%, respectively; adjusted mean difference: −0.08%; *P* = 0.578) (Figure 4B).

## 4 DISCUSSION

There is a growing body of RWE on glycaemic control and hypoglycaemia mirroring RCT data, but relatively few reports concerning medication persistence or discontinuation. Our data show differences in baseline demographics, clinical characteristics and clinical outcomes during follow‐up, in patients with T2D switching their basal insulin to Gla‐300 compared with those switching to other basal insulins. A greater proportion of patients who switched to Gla‐300 remained persistent throughout the follow‐up period. After adjusting for baseline characteristics, this analysis reports comparable effectiveness and a lower likelihood of hypoglycaemia in patients who switched to Gla‐300 compared with those who switched to other basal insulins.

Many of the symptoms of hypoglycaemia, such as weakness, dizziness and rapid heartbeat, may be particularly challenging for elderly individuals by adding to an already increased risk for falls, cognitive impairment and other complications.^18^, ^19^ Even among patients with T2D who initiate basal insulin, it is estimated that only 30% achieve their individual glycemic target, in part due to concerns around hypoglycaemia which may result in less timely and effective insulin titration.^20^, ^21^ It has also been shown that hypoglycaemia leads to poor adherence and clinical outcomes, concurrent with increased healthcare resource use.^22^, ^23^, ^24^ Our treatment discontinuation data for both cohorts are therefore in line with observed rates from other studies.

Differences in baseline demographics and clinical characteristics may guide physicians’ prescribing decisions. Although there was a statistically significant difference in age between the two groups, the actual numerical difference of only 2 years would suggest that age was an unlikely driver of treatment decisions. The lower CCI score, and lower use of inpatient and ER facilities, suggest that patients switching to Gla‐300 were healthier. The presence of comorbidities is known to be a major consideration for physicians considering initiating insulin therapy; however, it is unclear how general health might affect switching in patients already using basal insulin.^17^ Data from previous studies on insulin switching suggest that a higher burden of illness is a driver for switching between basal insulin analogs.^25^ It may be that in our cohort, the lower level of illness in Gla‐300 switchers is reflective of switching driven by the perceived advantages of the Gla‐300 rather than by problems with the current basal insulin therapy. Additionally, patients switching to Gla‐300 had higher baseline insulin doses.

Patients who switched to Gla‐300 had experienced fewer hypoglycaemic events at baseline than those who switched to another basal insulin. The substantially higher use of NPH insulin in the other switcher group is a possible explanation for this difference, as NPH insulin is associated with a higher rate of hypoglycaemia than Gla‐100.^26^, ^27^ It is notable that in a small retrospective study, concern regarding hypoglycaemia was one of the main reasons cited for switching to Gla‐300, a result somewhat in contrast to our own data.^10^

Patients switching to Gla‐300 were found to be more likely to remain persistent during follow‐up than those switching to other basal insulins. Although there were twice as many Gla‐300 switchers, our selection criteria were not preferential for Gla‐300 switchers over other switchers. Therefore, the prevalence of more Gla‐300 switchers during this time period may be a function of prescriber pattern or health plan characteristics. However, we did adjust for all patient characteristics.

The previous studies have demonstrated differences in treatment persistence following switching of basal insulin, with patients switching from Gla‐100 to insulin detemir being less persistent than those switching from insulin detemir to Gla‐100^26^, ^28^). Additionally, a positive correlation has been shown between specialist visits and medication treatment adherence.^29^ This combined with lower levels of hypoglycaemia seen in Gla‐300 switchers may contribute to better persistence, as patients who are concerned about hypoglycaemia are more likely to discontinue their insulin therapy. In addition, both patients who are not achieving their A1C targets, and their physicians, cite hypoglycaemia as a barrier to effective insulin titration.^30^

There are several other factors associated with Gla‐300 treatment that may predispose to better persistence, including flexibility of injection timing, single daily injections and lower injections volumes.^25^ Our study reinforces data from the EDITION trial series showing that Gla‐300 is associated with less hypoglycaemia than other basal insulins.^6^, ^7^, ^8^, ^9^ Patients in this cohort who switched to Gla‐300 were less likely to experience hypoglycaemia both at 3‐ and at 6‐month follow‐up compared with those who switched to other basal insulins. Similar results were found in a real‐world study that used propensity matching to harmonize baseline characteristics between groups, controlling for baseline confounders of hypoglycaemia.^11^

In the subgroup analysis of patients who had A1C measurements at baseline and during 3‐ to 6‐month follow‐up, glycaemic control was similar between the two switcher groups, a result which is in line with both the Phase III EDITION trial programme and a similar study comparing patients switching to Gla‐300 or other basal insulins.^6^, ^7^, ^8^, ^9^

### 4.1 Limitations

While the findings represent actual treatment patterns and outcomes outside the confines of clinical trials, several limitations should be noted.

Switching treatment regimen can be a complex decision, with both clinical and socioeconomic considerations; medical claims data do not reveal the reasons why patients and their providers switch basal insulins. Claims data capture the prescription and dispensing of medication, but not the consumption of medication. Consequently, prescription information may not reflect the actual drug use in real life. Additionally, claims data are collected for payment purposes and may have inherent limitations for clinical research, such as incomplete data available to assess hypoglycaemia frequency and glycaemic impact.

The study is likely to underreport hypoglycaemia, as only ICD‐9‐CM/ICD‐10‐CM codes associated with a healthcare encounter were captured. Data interpretation may also be affected by administrative diagnosis‐coding errors. Also, insulin amounts are difficult to accurately capture as the number of units of insulin originally prescribed is not always the actual number of units used by the patient, for several reasons, including up‐titration of insulin doses.

Despite the use of regression analysis to control for differences in baseline factors between patients, this observational retrospective database study is not able to infer causality. The follow‐up period for this study was relatively short; however, the long‐term comparative effectiveness of Gla‐300 and other basal insulins warrants further research. Furthermore, comparison of switching to a single insulin with switching to a group of insulins makes it difficult to interpret how therapeutic differences might affect outcomes and persistence.

## 5 CONCLUSIONS

Randomized trial data have demonstrated similar A1C values with reduced hypoglycaemia in patients previously treated with basal insulin who switched to the second‐generation basal insulin analog Gla‐300. Our data collected from a real‐world clinical setting show that patients who switched to Gla‐300 had better persistence and an associated lower risk of hypoglycaemia compared with patients who switched to other basal insulins; both treatments showed comparable glycaemic control in the subset of patients with available data. The reduced incidence and rate of hypoglycaemia experienced, particularly in the first 3 months after treatment switch, may have contributed to the improved persistence observed with Gla‐300. The successful early initiation of insulin may therefore be an important factor in the long‐term successful use of insulin.

## CONFLICT OF INTEREST

LX, YW and NV are employees of STATinMED Research; LZ and CN are employees and shareholders of Sanofi; LFM is a consultant for Sanofi Aventis, advisory board member for Sanofi Aventis, Novo Nordisk and Astra Zeneca and has received grant support from the American Diabetes Association.

## AUTHOR CONTRIBUTION

LZ, LX and YW designed the study. LX, YW and NV acquired the data. All authors interpreted the data and provided critical revisions to the manuscript.

## ETHICAL APPROVAL

This study was conducted in compliance with the ethics guidelines for research in humans as recorded in the Helsinki Declaration of 1964. Given the observational retrospective nature of this study, individual consent was not required after ensuring for anonymization of data.

## Data Availability

All data are included within the manuscript.
