# Supplementary material for: Hypoglycaemia and treatment patterns among insulin‐treated patients with type 2 diabetes who switched to insulin glargine 300 units/mL versus other basal insulin in a real‐world setting
Source: Endocrinol Diabetes Metab. 2019 Jun 14;2(3):e00073. doi: 10.1002/edm2.73 (PMC6613231; doi:10.1002/edm2.73)
Supplement: Supplementary file 1 [file EDM2-2-e00073-s001.docx]

**Supplementary material**

**Supplementary Table 1. Baseline Descriptive Characteristics of Gla-300 Switchers vs. Other Switchers (During the 6 months pre-index date).**

|  | **Gla-300 Switchers** | | **Other Switchers** | | | |
| --- | --- | --- | --- | --- | --- | --- |
|  | **N= 492** | | **N= 242** | | | |
|  | **N/Mean** | **%/SD** | **N/Mean** | **%/SD** | **p-value** | **Standardized Difference** |
| **Demographic Characteristics** | | | | | | |
| Female | 260 | 52.85% | 128 | 52.89% | 0.9904 | 0.09 |
| **Health Plan Type** |  |  |  |  |  |  |
| Indemnity | 0 | 0.00% | 0 | 0.00% |  | 0.00 |
| POS | 34 | 6.91% | 57 | 23.55% | <.0001 | 47.53 |
| HMO | 215 | 43.70% | 108 | 44.63% | 0.8116 | 1.87 |
| PPO | 27 | 5.49% | 11 | 4.55% | 0.5880 | 4.31 |
| EPO | 9 | 1.83% | 17 | 7.02% | 0.0003 | 25.42 |
| Others | 207 | 42.07% | 49 | 20.25% | <.0001 | 48.42 |
| **Payer Type** |  |  |  |  |  |  |
| Commercial | 58 | 11.79% | 88 | 36.36% | <.0001 | 59.91 |
| Medicare | 434 | 88.21% | 154 | 63.64% | <.0001 | 59.91 |
| **CCI** | 3.65 | 2.23 | 4.19 | 2.05 | 0.0019 | 24.84 |
| **Oral Anti-Diabetic Medications** | | | | | | |
| Any OAD | 304 | 61.79% | 148 | 61.16% | 0.8686 | 1.30 |
| Number of OADs | 1.04 | 1.04 | 1.00 | 1.01 | 0.6334 | 3.76 |
| Biguanide | 200 | 40.65% | 108 | 44.63% | 0.3046 | 8.04 |
| DPP-4 Inhibitors | 66 | 13.41% | 29 | 11.98% | 0.5871 | 4.29 |
| Meglitinide Derivatives | 7 | 1.42% | 3 | 1.24% | 0.8406 | 1.60 |
| Sulfonylureas | 116 | 23.58% | 61 | 25.21% | 0.6276 | 3.79 |
| Thiazolidinediones | 26 | 5.28% | 9 | 3.72% | 0.3494 | 7.54 |
| α-Glucosidase Inhibitors | 2 | 0.41% | 0 | 0.00% | 0.3206 | 9.03 |
| SGLT2 Inhibitors | 33 | 6.71% | 18 | 7.44% | 0.7144 | 2.85 |
| GLP-1 Agonist Use | 63 | 12.80% | 15 | 6.20% | 0.0063 | 22.64 |
| **Baseline Basal Use (Closest to the Index Date)** | | | | | | |
| NPH | 13 | 2.64% | 123 | 50.83% | <.0001 | 129.53 |
| Levemir | 134 | 27.24% | 30 | 12.40% | <.0001 | 37.84 |
| Lantus | 345 | 70.12% | 89 | 36.78% | <.0001 | 70.82 |
| **HBA1c and DACON** | | | | | | |
| Baseline HbA1c Values (A1C in 6m Baseline) | (N=492) | | (N=242) | |  | |
| Average Baseline HbA1c Values | 8.84 | 1.69 | 8.78 | 1.81 | 0.6211 | 3.84 |
| <7% | 59 | 11.99% | 29 | 11.98% | 0.9974 | 0.03 |
| ≥7% and <8% | 101 | 20.53% | 60 | 24.79% | 0.1893 | 10.18 |
| ≥8% and <9% | 139 | 28.25% | 58 | 23.97% | 0.2181 | 9.75 |
| ≥9% | 193 | 39.23% | 95 | 39.26% | 0.9941 | 0.06 |
| Baseline Basal insulin DACON (units/day) | 45.08 | 43.11 | 33.85 | 50.81 | 0.0033 | 23.82 |
| **Baseline Hypoglycemic Events** | | | | | | |
| Any Hypoglycemia | 58 | 11.79% | 38 | 15.70% | 0.1393 | 11.37 |
| Any Inpatient/ER Hypoglycemia | 9 | 1.83% | 11 | 4.55% | 0.0336 | 15.48 |
| Any Outpatient Hypoglycemia | 52 | 10.57% | 34 | 14.05% | 0.1681 | 10.59 |
| **Baseline Healthcare Utilizations** | | | | | | |
| Baseline HealthCare Utilizations |  |  |  |  |  |  |
| Any Inpatient Stay | 49 | 9.96% | 61 | 25.21% | <.0001 | 40.81 |
| Any ER Visit | 118 | 23.98% | 88 | 36.36% | 0.0004 | 27.18 |
| Any Endocrinology Visit | 139 | 28.25% | 54 | 22.31% | 0.0858 | 13.67 |

CCI, Charlson Comorbidity Index; DACON, daily average consumption; DPP-4, dipeptidyl peptidase-4; EPO, exclusive provider organization; GLP-1, Glucagon-like peptide 1; HMO, health maintenance organization; NPH, Neutral Protamine Hagedorn; OAD, Oral Antidiabetes Drug; POS, noncapitated point of service; PPO, preferred provider organization; SGLT2, sodium glucose co-transporter 2.

**Supplementary Table 2.** Patients who had A1c in 6 months baseline period.

| **Index Insulin** | **Other Switchers (N= 616)** | |
| --- | --- | --- |
|  | **N/Mean** | **%/Std** |
| **Lantus** | 259 | 42.05% |
| **Levemir** | 355 | 57.63% |
| **Tresiba** | 2 | 0.32% |

**Supplementary Table 3.** Patients who had A1c in follow-up (between 3-6 months post index date)

| **Index Insulin** | **Other Switchers (N= 242)** | |
| --- | --- | --- |
|  | **N/Mean** | **%/Std** |
| **Lantus** | 89 | 36.78% |
| **Levemir** | 153 | 63.22% |
